# Transcranial direct current stimulation in post-stroke sub-acute aphasia: study protocol for a randomized controlled trial

**DOI:** 10.1186/s13063-016-1505-z

**Published:** 2016-08-02

**Authors:** Kerstin Spielmann, W. Mieke E. van de Sandt-Koenderman, Majanka H. Heijenbrok-Kal, Gerard M. Ribbers

**Affiliations:** 1 Rijndam Rehabilitation Institute, PO Box 23181, 3001 KD Rotterdam, The Netherlands; 2 Department of Rehabilitation Medicine, Erasmus MC, University Medical Center Rotterdam, PO Box 2040, 3000 CA Rotterdam, The Netherlands

**Keywords:** Transcranial direct current stimulation, Aphasia, Stroke, Sub-acute

## Abstract

**Background:**

Transcranial direct current stimulation (tDCS) is a promising new technique to optimize the effect of regular Speech and Language Therapy (SLT) in the context of aphasia rehabilitation. The present study focuses on the effect of tDCS provided during SLT in the sub-acute stage after stroke. The primary aim is to evaluate the potential effect of tDCS on language functioning, specifically on word-finding, as well as generalization effects to verbal communication. The secondary aim is to evaluate its effect on social participation and quality of life, and its cost-effectiveness.

**Methods:**

We strive to include 58 stroke patients with aphasia, enrolled in an inpatient or outpatient stroke rehabilitation program, in a multicenter, double-blind, randomized controlled trial with two parallel groups and 6 months’ follow-up. Patients will participate in two separate intervention weeks, with a pause of 2 weeks in between, in the context of their regular aphasia rehabilitation program. The two intervention weeks comprise daily 45-minute sessions of word-finding therapy, combined with either anodal tDCS over the left inferior frontal gyrus (1 mA, 20 minutes; experimental condition) or sham-tDCS over the same region (control condition). The primary outcome measure is word-finding. Secondary outcome measures are verbal communication, social participation, quality of life, and cost-effectiveness of the intervention.

**Discussion:**

Our results will contribute to the discussion on whether tDCS should be implemented in regular aphasia rehabilitation programs for the sub-acute post-stroke population in terms of (cost-)effectiveness.

**Trial registration:**

Nederlands Trail Register: NTR4364. Registered on 21 February 2014.

**Electronic supplementary material:**

The online version of this article (doi:10.1186/s13063-016-1505-z) contains supplementary material, which is available to authorized users.

## Background

Aphasia is present in about 30 % of patients immediately after stroke [1]. In the first weeks and months, considerable recovery may occur; however, about 20 % are left with chronic deficits at 6 months post stroke [2, 3]. There is increasing support for the efficacy of Speech and Language Therapy (SLT) in order to diminish the language and communication deficits that people with aphasia encounter [4]; however, it remains a challenge to optimize the effect of aphasia therapy.

Transcranial direct current stimulation (tDCS) is a promising new technique to optimize the effect of regular SLT in the context of aphasia rehabilitation [5]. It is safe and easy to apply and has limited side effects [6]. tDCS modulates cortical excitability by delivering weak electric currents to the cortex via two electrodes applied to the skull [7]. The effect of tDCS depends on the polarity of the electrodes: anodal tDCS enhances neuronal excitability while cathodal tDCS diminishes neuronal excitability. This effect is related to a change in the resting membrane potential. Anodal tDCS leads to de-polarization, increasing the chance for an action potential, and cathodal tDCS leads to hyper-polarization [8, 9]. tDCS is also related to neuroplasticity. Specifically, processes like long-term potentiation and secretion of brain-derived neurotrophic factor (BDNF) are associated with tDCS application [10]. The potential benefits of tDCS applied during SLT have been described since 2008 [5, 11–17]. However, these studies have some methodological limitations such as small sample size and lack of randomization.

The application of tDCS to enhance the effect of SLT is associated with the notion that tDCS may have a role in rebalancing the activity of both hemispheres post stroke. Language processing is strongly lateralized to the left hemisphere (LH), at least in right-handed healthy individuals [18–21]. After LH damage and aphasia, the right hemisphere (RH), may show increased activity. Whether this increased activity in the RH is adaptive or maladaptive, is an unresolved issue [22–24]. However, most studies indicate that, in the long term, LH perilesional recruitment is associated with better aphasia recovery, while RH recruitment is related to incomplete recovery [25–27]. In line with these observations, most studies use tDCS as a tool to promote LH perilesional recruitment.

Across studies, different electrode configurations are used to promote LH perilesional recruitment. In some studies anodal tDCS [13, 15, 16] is applied either to the left inferior frontal gyrus (IFG: Broca’s area) or to the left superior temporal gyrus (Wernicke’s area), while other studies use cathodal tDCS to inhibit the RH homolog areas, so as to disinhibit the LH [14, 28]. Few studies use an individual approach for electrode configurations [11, 29]. Anodal tDCS to the left IFG, with the cathode placed on the contralateral supra-orbital region, is the most common configuration, which has been supported by studies investigating this further with functional magnetic resonance imaging (fMRI) [30, 31] and computer modeling [32]. Predominantly, tDCS studies choose word-finding therapy as the behavioral treatment component. Irrespective of electrode configurations, studies point to an additional effect of tDCS on language functioning, when combined with SLT [5, 11–17, 29].

Studies evaluating tDCS in sub-acute aphasia rehabilitation are limited [26]. Evaluating the potential of tDCS in patients with sub-acute aphasia is important, as the larger proportion of language treatment for stroke patients is provided in the sub-acute phase, during the first weeks and months post stroke. During these first months, the recovery rate is highest [33].

Therefore, the aim of the present study is to investigate the effect of tDCS in sub-acute stroke patients with aphasia who are enrolled in regular stroke rehabilitation services. In line with studies applying tDCS in the chronic stage, we use the most common electrode configuration, i.e., anodal tDCS over the left IFG as compared to sham-tDCS, in combination with disorder-oriented aphasia therapy, aimed at word-finding. The cathode is placed on the contralateral supra-orbital region.

### Objective

The present study focuses on the effect of tDCS provided during SLT in the sub-acute stage after stroke. The primary aim is to evaluate the effect of tDCS on language functioning. The primary outcome measure is word-finding. Secondary outcome measures are verbal communication, social participation, quality of life, and the cost-effectiveness of the intervention.

## Methods

### Study design and procedure

**Fig. 1:**
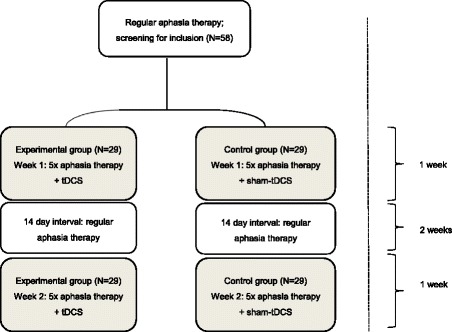
Study design with two separate intervention weeks

The study is a multicenter, double-blind, randomized controlled trial with two parallel groups and 6-month follow-up. Patients will participate in two separate intervention weeks, with a pause of 2 weeks in between, in the context of regular aphasia rehabilitation (see Fig. 1). During each intervention week, regular SLT sessions are replaced by daily 45-minute sessions of word-finding therapy, combined with either anodal tDCS over the left IFG (1 mA, 20 minutes; experimental condition) or sham-tDCS over the same region (control condition). The cathode is placed on the contralateral supra-orbital region. To our knowledge, a parallel design with two separate intervention weeks has not been used before in the tDCS literature. This design allows measurements before and after each intervention week, thus providing information on the recovery pattern over time within one subject.

All other therapies in the participant’s stroke rehabilitation program, such as physical therapy or occupational therapy remain unchanged and are offered following the stroke rehabilitation protocol of each participating rehabilitation center.

### Setting and study population

Stroke patients with aphasia, who are receiving regular aphasia therapy, will be screened for eligibility and start the intervention between 3 weeks and 3 months after their stroke. These patients are enrolled in regular stroke rehabilitation (inpatient and outpatient services) in four rehabilitation centers in the Netherlands: Rijndam Rehabilitation (Rotterdam), Libra Rehabilitation (Tilburg and Eindhoven), Revant Rehabilitation (Breda) and De Hoogstraat Rehabilitation (Utrecht). Table 1 lists the inclusion and exclusion criteria. We strive to include 58 patients, based on a power analysis (see section Data analysis). Before inclusion, all participants need to sign the informed consent form. Patient information is provided orally as well as in written form, with extra versions in an aphasia-friendly format. This study has been approved by the Medical Ethics Committee (MEC) of the Erasmus MC, University Medical Center Rotterdam. The researcher will report serious adverse events (SAE) to the MEC and SAEs are handled according to the WMO (“Wet Medisch-wetenschappelijk Onderzoek”), the Dutch law for medical scientific research. tDCS is known to be a safe intervention with minimal side effects [6]. Participants who develop post-stroke epileptic seizures before the end of the 4-week intervention will be withdrawn from the intervention, but not from the study; all assessments will be completed (intention-to-treat analysis).

**Table 1:** Inclusion and exclusion criteria

Inclusion criteria
1. Aphasia after stroke
2. Less than 3 months post onset
3. Age 18–80 years
4. Near-native speaker of Dutch
5. Being right-handed
6. Able to participate in intensive therapy
Exclusion criteria
1. Subarachnoid hemorrhage
2. Prior stroke resulting in aphasia
3. Brain surgery in the past
4. Epileptic activity in the past 12 months
5. Premorbid (suspected) dementia
6. Premorbid psychiatric disease affecting communication (for example, personality disorder)
7. Excessive use of alcohol or drugs
8. Presence of a cardiac pacemaker
9. Severe non-linguistic cognitive disturbances impeding language therapy
10. Global aphasia, defined as Shortened Token Test score <9 [50] and score 0 on the Aphasia Severity Rating Scale [41]
11. Severe Wernicke’s aphasia, defined as Shortened Token Test score <9 and score 0–1 on the Aphasia Severity Rating Scale
12. Residual aphasia, defined as Shortened Token Test score >28 and score 4–5 on the Aphasia Severity Rating Scale and Boston Naming Test score >150 [40]

### Randomization and blinding

Randomization is stratified per center of inclusion. To randomize participants to the experimental or control condition, we use a list of five-number codes, provided by the manufacturer of the stimulation device. Half of these codes activate the device to deliver anodal tDCS (experimental condition) and half of these codes deliver sham-tDCS (control condition). Codes are block randomized with a block size of four on the basis of a computer-generated sequence and then concealed in consecutively numbered, sealed, opaque envelopes. The envelope is opened at the start of the first intervention session. The participant’s unique five-number code is used to start the tDCS device, which then provides either real stimulation or sham as related to the code. The randomization and the preparation of the envelopes are done by a researcher (MH) of our research team, who is not involved in assessments and training of the patient. The key to the five-number codes is also kept by this researcher (MH). Consequently, the participants, their SLTs and the trial coordinator are blinded to treatment condition.

### Intervention

In each intervention week, regular SLT sessions are replaced by daily 45-minute sessions of word-finding therapy, combined with either anodal tDCS over the left IFG (1 mA, 20 minutes; experimental condition) or sham-tDCS over the same region (control condition). Therapy is provided by speech and language therapists of the participating centers. The cathode is placed on the contralateral supra-orbital region. The intensity of 1 mA tDCS for 20 minutes and the frequency of five sessions per week is in line with most studies applying tDCS in the chronic stage [11, 13–16]. tDCS is combined with word-finding therapy, because most people with aphasia have word-finding difficulties [34]. The word-finding therapy protocol is based on Cueing Hierarchy Therapy [35]. The participant’s task is to name a picture and, based on the protocol, the therapist uses cueing techniques to help the participant to retrieve and produce the target word correctly. The cue of low stimulus power is presented first, followed by increasingly powerful cues until the correct word is retrieved and produced. Basically, the following cueing hierarchy is used: (1) “What is this?”(e.g., show picture of a tree), (2) “Can you write the word down?”, (3) graphemic cueing (e.g., provide the number of letters), (4) phonological cueing (e.g., provide the first sound, /t/), (5) semantic associations (e.g., “can you tell where you can find these”), (6) therapist says the word (e.g., “tree”), (7) repetition of the target word.

As the relative power of the cues differs across participants with aphasia, the exact cueing hierarchy is personalized. For each picture, even if the picture is named without cues, the participant is encouraged to write or copy the correct word form or, in case of inability to write, to perform an anagram task. The rationale for incorporating production of the written word, is the evidence that activating the written word has a beneficial effect on retrieving spoken words [36].

To ensure relevance of the training material for each participant, stimuli are selected on the basis of individual naming performance at baseline using the European Data Bank (EDB) for oral picture naming [37]. The first 68 items the participant is unable to name correctly within 20 s are selected. These items are divided in two sets of 34 items, matched for word length and word frequency: a therapy set, trained during the word-finding therapy, and a control set, to evaluate generalization effects to untrained items. In the first session 10 items are trained. Then, during each session new items are added, with eight new items in the second session; six new items in the third and fourth session, and four new items in the final session. For the second intervention week a new training set is selected in the same way.

### tDCS

The DC Stimulator PLUS (produced by Eldith), certified as a medical device, class IIa, by the European Union Notified Body 0118 (CE 118), is used in the authorized form. Two electrodes (5 x 7 cm) are placed on the head and fixed with elastic tape; electrode placement is guided by the international 10-10 electroencephalogram (EEG) system and previous studies [15, 38, 39]. The anode is placed on the left IFG, localized as F5, and the cathode is placed on the contralateral supra-orbital region, localized as Fp2. Participants in the experimental condition receive active stimuli of 1 mA during 20 minutes. The stimulation is automatically activated with a fade in of 15 s and after 20 minutes the stimulation is automatically deactivated, with a fade out of 15 s. Participants in the control condition receive inactive stimulation (sham-tDCS), i.e., at first the stimulation is automatically activated with a fade in of 15 s, and then the stimulation is deactivated after 30 s, with a fade out of 15 s. Both the patient and the therapist are blinded for stimulation condition. The electrodes are not removed until completion of the 45-minute therapy session.

### Measurement instruments

Table 2 gives an overview of the measurement instruments being used. The primary outcome measure is the score on the Boston Naming Test (BNT) [40], to assess picture-naming. Secondary outcome measures are chosen to evaluate generalization of treatment effects to verbal communication: the Aphasia Severity Rating Scale (ASRS) [41] to assess spontaneous speech and the Amsterdam Nijmegen Everyday Language Test (ANELT) [42] as a measure for verbal communication in everyday life. Other secondary outcome measures are chosen to evaluate quality of life (EuroQol-5D, [43]; Stroke and Aphasia Quality Of Life questionnaire (SAQOL), [44, 45]), social participation (Community Integration Questionnaire, [46]), and cost-effectiveness (Cost Analysis Questionnaire, [47–49]).

**Table 2:** Measurement instruments

Language and communication tests
Boston Naming Test (BNT) [40]
Aphasia Severity Rating Scale (ASRS) [41]
Amsterdam Nijmegen Everyday Language Test (ANELT) [42]
Shortened Token Test [50]
Quality of life questionnaires
EuroQol-5D (EQ-5D) [43]
Stroke and Aphasia Quality Of Life questionnaire (SAQOL) [44, 45]
Other tests
Community Integration Questionnaire (CIQ) [46]
Cost Analysis Questionnaire [47–49]
Barthel Index [51]
Edinburgh Handedness Inventory
Wong-Baker Faces pain rating scale [52]

**Fig. 2:**
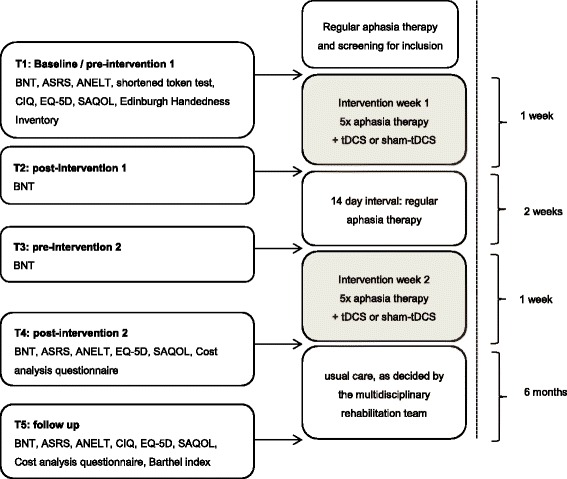
Measurement instruments and test moments

The primary outcome measure, BNT, is assessed before and after each intervention week (T1, T2, T3, T4) and at 6 months’ follow-up (T5); see Fig. 2. The secondary outcome measures are assessed before the first intervention week and after the second intervention week (T1, T4) and at 6 months (T5). The EuroQol-5D (EQ-5D) and the Cost Analysis Questionnaire are used to evaluate cost-effectiveness during the 4-week intervention period and during the follow-up period.

Baseline assessments (T1) include handedness (Edinburgh Handedness Inventory), aphasia severity (Shortened Token Test) [50] and overall functioning (Barthel Index) [51]. To register potential adverse effects, participants are asked to rate their discomfort immediately after each therapy session on the Wong-Baker Faces pain rating scale, a visual analog scale designed for patients with limited verbal skills [52].

To summarize the present study protocol, please see Additional file 1 for an overview of enrollment, intervention and measurement instruments, and see Additional file 2 for an overview of the Standard Protocol Items: Recommendations for Interventional Trials (SPIRIT) checklist.

### Sample size

The power calculation is based on the results of a randomized controlled trial by Baker et al. [11] including stroke patients in the chronic phase. In this study the group of aphasia patients trained with tDCS improved 2.1 points more than a sham-control group on a picture-naming test. Cohen’s *d* effect size was 0.22, which is equal to a Cohen’s *f* of 0.11. For the present study we calculated that, using a study design with two groups and four repeated measurements, a within-patient correlation of 0.75, an alpha of 0.05, a power of 0.80 and a Cohen’s *f* effect size of 0.11, we need a total group of 58 patients (29 patients in each treatment arm).

### Data analysis

Once randomized, each patient will be analyzed in the group to which they were assigned, independent of potential drop-out or compliance to the protocol, according to the intention-to-treat principle. Potential baseline differences between the groups will be tested using independent *t* tests for continuous variables, the Mann-Whitney *U* test for ordinal variables, and chi-square tests for categorical variables.

Outcomes of the measures over time will be compared for the experimental condition versus the control condition using repeated measurements analysis. This analysis takes into account the correlation of repeated measurements within the same patients and it can handle missing data, assuming that data are missing at random. The dependent variable is the outcome measure and the independent variables are time and group assignment and the interaction between these variables. In these analyses, adjustments can be made for potentially confounding variables that could be unequally distributed over the groups despite the randomization procedure.

To evaluate cost-effectiveness, direct (para)medical costs and the total costs of all separate treatments by health care providers during the intervention period will be summed, as well as the costs of the facilities and materials used for these treatments. In addition, the non-medical costs, such as productivity loss, will be calculated. The incremental cost-effectiveness ratio will be calculated by dividing the difference in total costs by the difference in Quality-adjusted Life Years (QALYs), based on the EQ-5D. A net health-benefit analysis will be used to relate the costs to the benefit. We assume that the economic value of 1 life year in good health amounts to 25,000–50,000 €. The economic evaluation will be performed following the Dutch guidelines [53].

## Discussion

The present study focuses on the effect of tDCS provided during SLT in the sub-acute stage after stroke. The primary aim is to evaluate the potential effect of tDCS on language functioning, specifically on word-finding, as well as generalization effects to verbal communication. The secondary aim is to evaluate its effect on social participation and quality of life, and to evaluate the cost-effectiveness of this intervention.

In line with studies applying tDCS in the chronic stage, we use the most common electrode configuration, i.e., anodal tDCS over the left IFG as compared to sham-tDCS, in combination with disorder-oriented aphasia therapy, aimed at word-finding. The application of tDCS (1 mA for 20 minutes) and the frequency is also chosen in line with studies applying tDCS in the chronic stage. However, the discussion of what may be the optimal electrode configuration and what the optimal stimulation intensity and frequency is, is still ongoing. Regarding the optimal electrode configuration, individual factors such as lesion size and the relative contribution of the RH and the LH and its relation to aphasia recovery, may lead to individual variability in response to tDCS. However, recent fMRI and computer modeling studies find that applying anodal tDCS on the left IFG [30–32] may be a suitable approach.

We expect that tDCS will enhance the speed of language recovery, resulting in improved communication, quality of life and participation – associated with decreased rehabilitation consumption and cost reduction. If we find that tDCS enhances the effect of SLT in an early phase provided that adverse effects are limited at this stage post stroke, and if it is found to be cost-effective, tDCS may be implemented in regular aphasia rehabilitation programs for the sub-acute post-stroke population.

### Trial status

The inclusion of the study started on 17 April 2014 and on 2 May 2016 we had included 44 participants. We expect that the inclusion and the follow-up measurements will be completed in April 2017.

## Abbreviations

ANELT, Amsterdam Nijmegen Everyday Language Test; ASRS, Aphasia Severity Rating Scale; BDNF, brain-derived neurotrophic factor; BNT, Boston Naming Test; CIQ, Communication Integration Questionnaire; EDB, European Data Bank; EQ-5D, EuroQol-5D; IFG, inferior frontal gyrus; LH, left hemisphere; QALYs, Quality-adjusted Life Years; RH, right hemisphere; SAQOL, Stroke and Aphasia Quality Of Life; SLT, Speech and Language Therapy; tDCS, transcranial direct current stimulation.

## Additional files

Additional file 1: Figure S1. Schedule of enrollment, interventions, and assessments. (DOC 54 kb) Additional file 2: SPIRIT 2013 Checklist: recommended items to address in a clinical trial protocol and related documents. (DOC 121 kb)
