# Supplementary material for: Transcranial direct current stimulation in post-stroke sub-acute aphasia: study protocol for a randomized controlled trial
Source: Trials. 2016 Aug 2;17:380. doi: 10.1186/s13063-016-1505-z (PMC4970230; doi:10.1186/s13063-016-1505-z)
Supplement: Additional file 1: Figure S1. — Schedule of enrollment, interventions, and assessments. (DOC 54 kb) [file 13063_2016_1505_MOESM1_ESM.doc]

Additional file 1. Schedule of enrolment, interventions, and measurement instruments.

|  |  | | | | | | | |
| --- | --- | --- | --- | --- | --- | --- | --- | --- |
|  | **Enrolment** | **Allocation** | **Close-out** | |  | | | |
| **TIMEPOINT**** | ***-t_1_*** | **0** | ***T1*** | ***T2*** | | ***T3*** | ***T4*** | ***T5 (6 months followup)*** |
| **ENROLMENT:** |  |  |  |  | |  |  |  |
| **Eligibility screen** | X |  |  |  | |  |  |  |
| **Informed consent** | X |  |  |  | |  |  |  |
| **Allocation** |  | X |  |  | |  |  |  |
| **INTERVENTIONS:** |  |  |  |  | |  |  |  |
| ***Aphasia therapy + tDCS*** |  |  |  |  | |  |  |  |
| ***Aphasia therapy + sham tDCS*** |  |  |  |  | |  |  |  |
| ***[List other study groups]*** |  |  |  |  | |  |  |  |
| **MEASUREMENT INSTRUMENTS:** |  |  |  |  | |  |  |  |
| ***Boston naming Test*** |  |  | X | X | | X | X | X |
| ***ASRS*** |  |  | X |  | |  | X | X |
| ***ANELT*** |  |  | X |  | |  | X | X |
| ***EQ-5D*** |  |  | X |  | |  | X | X |
| ***CIQ*** |  |  | X |  | |  |  | X |
| ***SAQOL*** |  |  | X |  | |  | X | X |
| ***Cost analysis questionnaire*** |  |  |  |  | |  | X | X |
| ***Shortened Token Test*** | X |  |  |  | |  |  |  |
| ***Edinburgh Handedness Inventory*** | X |  |  |  | |  |  |  |
